# Novel mutant mouse line emphasizes the importance of protein kinase C theta for CD4^+^ T lymphocyte activation

**DOI:** 10.1186/s12964-019-0364-0

**Published:** 2019-05-28

**Authors:** Kerstin Siegmund, Nikolaus Thuille, Nina Posch, Friedrich Fresser, Michael Leitges, Gottfried Baier

**Affiliations:** 1 Department for Pharmacology and Genetics, Medical University Innsbruck, Division of Translational Cell Genetics, Peter Mayr Str. 1a, A-6020 Innsbruck, Austria; 2 Biotechnology Center of Oslo, Oslo, Norway

**Keywords:** T lymphocyte signaling and activation, Protein kinase C theta (PKCθ), Interleukin- 2 (IL-2), Regulatory T cell development

## Abstract

**Background:**

The protein kinase C theta (PKCθ) has an important and non-redundant function downstream of the antigen receptor and co-receptor complex in T lymphocytes. PKCθ is not only essential for activation of NF-κB, AP-1 and NFAT and subsequent interleukin-2 expression, but also critical for positive selection and development of regulatory T lymphocytes in the thymus. Several domains regulate its activity, such as a pseudosubstrate sequence mediating an auto-inhibitory intramolecular interaction, the tandem C1 domains binding diacylglycerol, and phosphorylation at conserved tyrosine, threonine as well as serine residues throughout the whole length of the protein. To address the importance of the variable domain V1 at the very N-terminus, which is encoded by exon 2, a mutated version of PKCθ was analyzed for its ability to stimulate T lymphocyte activation.

**Methods:**

T cell responses were analyzed with promoter luciferase reporter assays in Jurkat T cells transfected with PKCθ expression constructs. A mouse line expressing mutated instead of wild type PKCθ was analyzed in comparison to PKCθ-deficient and wild type mice for thymic development and T cell subsets by flow cytometry and T cell activation by quantitative RT-PCR, luminex analysis and flow cytometry.

**Results:**

In cell lines, the exon 2-replacing mutation impaired the transactivation of interleukin-2 expression by constitutively active mutant form of PKCθ. Moreover, analysis of a newly generated exon 2-mutant mouse line (PKCθ-E2^mut^) revealed that the N-terminal replacement mutation results in an hypomorph mutant of PKCθ combined with reduced PKCθ protein levels in CD4^+^ T lymphocytes. Thus, PKCθ-dependent functions in T lymphocytes were affected resulting in impaired thymic development of single positive T lymphocytes in vivo. In particular, there was diminished generation of regulatory T lymphocytes. Furthermore, early activation responses such as interleukin-2 expression of CD4^+^ T lymphocytes were significantly reduced even though cell viability was not affected. Thus, PKCθ-E2^mut^ mice show a phenotype similar to conventional PKCθ-deficient mice.

**Conclusion:**

Taken together, PKCθ-E2^mut^ mice show a phenotype similar to conventional PKCθ-deficient mice. Both our in vitro T cell culture experiments and ex vivo analyses of a PKCθ-E2-mutant mouse line independently validate the importance of PKCθ downstream of the antigen-receptor complex for activation of CD4^+^ T lymphocytes.

**Electronic supplementary material:**

The online version of this article (10.1186/s12964-019-0364-0) contains supplementary material, which is available to authorized users.

## Background

Signaling events downstream of the antigen-receptor/costimulatory receptor complex regulate many aspects of T lymphocyte physiology such as thymic development, proliferation, survival and cytokine expression. Upon ligand binding to the T cell receptor (TCR), several cytoplasmic molecules are recruited to the immunological synapse (IS) formed between T lymphocyte and antigen-presenting cell (APC), where they get activated and propagate the signal. One of these downstream signaling molecules is the gamma1 isoform of phospholipase C (PLCγ1). It catalyzes the formation of the second messenger inositol-(1,4,5)-trisphosphate (InsP3) and diacylglycerol (DAG), leading to the initiation of calcium-influx and activation of DAG-binding kinases such as serine/threonine protein kinase C (PKC), respectively. PKCtheta (PKCθ), a member of the novel Ca^2+^-independent PKC subfamily, is rapidly recruited to the IS upon T cell receptor (TCR) ligation [1, 2]. Its importance for T cell activation and thus T cell-mediated processes has been shown particularly by studies using PKCθ-deficient mice [3, 4]. According to these studies, mice deficient in PKCθ not only show a defect in thymic positive selection but are also severely impaired in peripheral T cell activation. Thus, using these knockout mouse models PKCθ has been shown to be essential for production of interleukin-2 (IL-2) and proliferation induced by stimulation via the TCR/costimulatory receptor complex [3, 4]. Moreover, results obtained with the Jurkat T cell line expressing recombinant PKCθ mutants support the findings on the essential role of PKCθ in T cell activation processes [5, 6]. In particular, at a molecular level in T cells, PKCθ contributes to the activation of the transcription factors NFAT, AP-1 and NF-κB, all of which have binding sites in the IL-2 promotor and are mandatory for its expression [3–8]. Therefore, the novel PKC isoenzyme PKCθ regulates multiple signaling pathways critical for T cell activation.

**Fig. 1:**
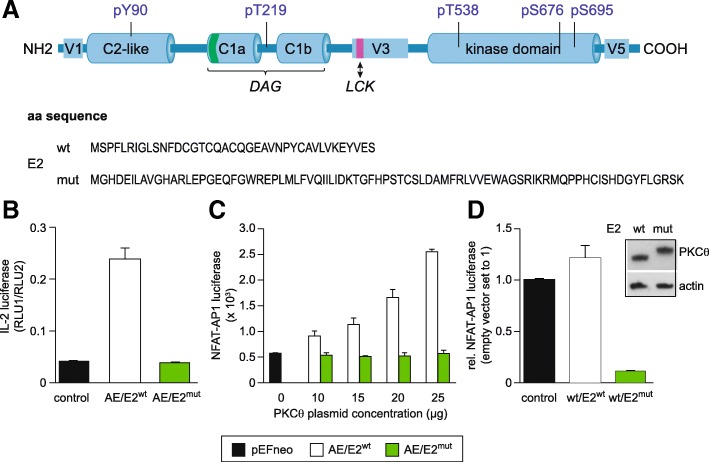
The PKCθ-E2 replacement mutant shows impaired IL-2 transactivation in Jurkat T cells. **a** Schematic depiction of the domain structure of PKCθ and the wild type (wt) and mutated (mut) aa sequence of the targeted exon 2 (E2). **b** IL-2 promoter luciferase reporter assay performed with Jurkat T cells transfected with the constitutively active mutant PKCθ A148E bearing either the wt or mutated E2 sequence – as indicated. Transfection with the empty vector was used as a control. Transfected cells were stimulated overnight with the calcium ionophore ionomycin. **c** NFAT-AP1-dependent promoter luciferase reporter assay using different concentrations of the PKCθ expression plasmids as indicated in the figure was performed similar to the assay shown in (b). **d** NFAT-AP1-dependent promoter luciferase reporter assay performed with HEK-293T cells transfected either with empty vector, PKCθ-E2^wt^ or PKCθ-E2^mut^. One day after transfection cells were stimulated overnight with PDBu/ionomycin. Expression of recombinant PKCθ (wt or E2^mut^) and endogenous actin was analyzed by immunoblot. The bar graph summarizes results of 3 independent experiments (mean ± SEM) and immunoblots of one experiment are shown

All members of the novel class of PKC isoenzymes (nPKC) share a common multi-modular structure. Besides the catalytic kinase domain at the C-terminus mediating its enzymatic function, they comprise a tandem DAG-binding C1 domains (C1a and C1b) involved in membrane recruitment, and a C2-like domain participating in protein-protein interaction (Fig. 1). Activation of nPKC is regulated by DAG, protein-protein interaction and additionally, by trans- as well as auto-phosphorylation events (reviewed in [9]). Furthermore, the so-called pseudo-substrate region, located within the C1a domain, resembles PKC substrates and forms an auto-inhibitory loop to keep the kinase in a closed, inactive conformation. Release from auto-inhibition is initiated after DAG-binding and recruitment to the plasma membrane. It is noteworthy that mutations that disrupt this intra-molecular interaction, such as the phosphomimic Ala to Glu mutation at aa residue 148 of PKCθ, generate a constitutively active form of this enzyme [5]. Besides the well-conserved domains within the (sub-)family mentioned above, PKC isoenzymes have distinctly different regions, and it has been suggested that these variable regions contribute to isoenzyme-selective functions [10]. A proline-rich motif within the V3 domain, for instance, mediates interaction between PKCθ and CD28 via LCK, and is essential for recruitment of PKCθ to the IS [11]. Moreover, the V5 domain, which is located C-terminal of the catalytic domain of classical and novel PKCs and shares hardly any sequence homology between the PKC isoenzymes, contains the highly conserved turn and hydrophobic phosphorylation motifs involved in regulation of enzymatic activity by stabilizing the catalytic core structure in a position allowing high-affinity interactions of ATP and its substrate [12]. Also the very distal portion of the V5 domain not comprising the conserved phosphorylation sites seems to be critical for catalytic function as shown for PKCα [13]. Additionally, it has been described that this domain is responsible for distinct subcellular localization patterns (reviewed in [14]). However, to our knowledge, the functional importance of the very N-terminal variable region (designated V1) of PKCθ has not been analyzed so far. Therefore, we addressed this research question by expressing exon 2/V1-domain exchange mutants in cell lines (Jurkat T cells and HEK-293T cells) as well as in a genetically modified mouse line (PKCθ-E2^mut^). Our results show that this mutation impairs PKCθ-mediated activation of T cells, such as the expression of IL-2, in both systems. Moreover, thymic development was impaired, with particularly reduced Treg frequency in vivo. In this regard, PKCθ-E2^mut^ mice are a phenocopy of PKCθ-deficient mice, which we analyzed in parallel. Mechanistically, the E2-mutation leads to reduced expression levels of PKCθ in murine T cells, generating an PKCθ hypomorphic murine mouse line.

## Materials and methods

### Mice

The conventional PKCθ knockout mouse line was as described previously [4]. The PKCθ-E2^mut^ line was generated in collaboration with Dr. Michael Leitges of The Biotechnology Centre of Oslo, Norway. Briefly, 12–16-weeks-old mice were used for the experiments shown. All mice were backcrossed to C57BL/6 for at least eight generations. Wt littermates were used as control mice. Genotyping was performed by PCR using the following primers: common forward primer: GCAGACCCAGACCATTCCCTAG (fwd) and reverse primer for prkcq-wt: CGCATTCGTCTACATGATAACCGAC; for prkc-ko: GGTAGTCTCGGATGGTTGAGGG; for prkc-E2mut: AGGATCTCCTGTCATCTCACCTTGCTCCTG; and the following thermocycler protocol: 30 cycles of 95 °C for 30 s, 64 °C for 30 s and 72 °C for 2 min. All mouse lines were housed under specific pathogen-free conditions. The animal experiments were conducted in accordance with the Austrian Animal Welfare Law and Animal Experimental Act (BGBI No. 501/1988 and BGBI. No. 114/2012), and were approved by the Committee of the Animal Care of the Austrian Federal Ministry of Science and Research (BM:WFW-66.011/0064-WF/V/3b/2016).

### Jurkat T cell and HEK-293T culture, transfection and luciferase assay

Jurkat T-Ag cells were maintained in RPMI medium (Biochrom) supplemented with 10% heat-inactivated FCS (Biochrom). Transient transfection of cells was performed by electroporation in a BTX-T820 ElectroSquarePorator (ITC, Biotech) apparatus using predetermined optimal conditions (2 × 10^7^ cells at 450 V/cm, 5 pulses of 99 ms). HEK-293T cells were cultured in DMEM medium supplemented with 10% heat-inactivated FCS, 2 mM L-Glutamine and 1% penicillin plus streptomycin (all Biochrom). For transient expression of PKCθ constructs, HEK 293 T cells were transfected using TurboFect™ transfection reagent (Thermo scientific) according to the manufacturer’s instructions.

One day after transfection the cells were stimulated overnight with the calcium ionophore ionomycin (200 ng/ml, Sigma) and the phorbol ester Phorbol 12,13-dibutyrate (PDBu; 10 ng/ml, Sigma) or with ionomycin alone in case of the PKCθ (A148E) constructs. For the reporter gene expression assays, the cells were transfected with the indicated reporter and expression constructs and measured as previously described [15]. PKCθ constructs have been cloned into the pEF1a-IRES-Neo vector (EF1a promoter driving expression). The renilla expression plasmid was obtained from Promega, the NFAT/AP-1 luciferase plasmid was a gift from Anjano Rao and the IL-2 luciferase plasmid was from Addgene. NFAT/AP-1 enhancer or minimal human IL***-***2 promoter reporter luciferase assays were performed using the Dual-Luciferase Reporter Assay System from Promega and luminescence was measured with a PHERAstar FS (BMG labtech).

### Thymocyte and splenocyte isolation, CD4^+^ T cell sorting and in vitro stimulation

Single cell suspensions of lymph nodes, spleens and thymi were prepared by mechanical disintegration using metal sieves or 100 μM cell strainers (Falcon), followed by the removal of erythrocytes by lyses (Mouse Erythrocyte Lysing Kit, R&D Systems). After a washing step with PBS/0.5% BSA/2 mM EDTA, viable cell counts were determined with a LUNA Automated Cell Counter (Logos Biosystems). CD4^+^ T cells were sorted negatively (untouched) by MACS technology using a CD4^+^ T Cell Isolation (130–090-860) kit together with pre-separation filters, LS columns and a QuadroMACS Separator (all Miltenyi Biotec) according to the manufacturer’s instructions. The sort purity was checked by flow cytometry. Cell counts were adjusted to 2 × 10^6^/ml complete RPMI 1640 medium (supplemented with 10% heat-inactivated FCS; Biochrom), 2 mM L-glutamine (Biochrom), 1% penicillin plus streptomycin (Biochrom), 10 μM 2-mercaptoethanol (Sigma), MEM nonessential amino acids (Sigma) and 1 mM sodium pyruvate (Sigma). Sorted CD4+ T cells were stimulated with plate-bound anti-CD3 (5 μg/ml, clone 2C11, produced in house) and soluble anti-CD28 (1 μg/ml, clone 37.51; BD Biosciences) antibodies. For qRT-PCR analyses, cells were harvested after 24 h; for Luminex analyses, the supernatant was harvested at 24 and 48 h of culture. For overnight analyses of viability and activation stimulation, single cell suspensions of spleen and lymph nodes were stimulated with anti-CD3 and anti-CD28 antibodies (10 and 5 μg/ml) for 18 h. For intracellular IL-2 staining, cells were stimulated with 5 and 1 μg/ml anti-CD3 and anti-CD28 in the presence of GolgiPlug (BD Biosciences) for 7 h.

### Flow cytometry and luminex technology

Surface staining (including preincubation with FcR block; anti CD16/32 (BD Biosciences) was performed as described previously [16]. The following antibodies were used: CD3 Pacific Blue, CD3 PE-Cy7, CD3 FITC, CD8 PerCP-Cy5.5, CD4 V500, CD4 APC, CD44 PE-Cy7, CD62L APC-Cy7, CD69 FITC, CD25 PE (all BD Biosciences). For viability analyses, cells were stained with Annexin V-PE (BD Biosciences) together with antibodies in HBBS/2%FCS (both Biochrom); 7AAD (eBioscience) was added approximately 5 min before the sample was acquired at the flow cytometer. For the staining of intracellular antigens, cells were fixed and subsequently permeabilized to the staining of surface antigens. The FoxP3 PE staining buffer set (eBiosciences) was used for the detection of Foxp3. For IL-2 staining (IL-2 PE, BD Biosciences), fixation buffer and intracellular staining permeabilization wash buffer (Biolegend) were used. Data were acquired on a FACSCalibur (CellQuest, BD Biosciences) or FACSVerse (FACSuite, BD Biosciences) and analyzed with FlowJo software. The concentration of secreted IL-2 in the culture supernatants was measured by Luminex xMAP Technology using a Bio-Plex Pro Mouse Cytokine IL-2 Set (No. 171G5003M), according to the manufacturer’s instructions, on a Bio-Plex suspension array system (all Bio-Rad).

### RNA extraction, cDNA synthesis and real-time quantitative RT-PCR

Total RNA was isolated using the RNeasy Mini Kit (Qiagen), and reverse transcription was performed with the Omniscript Kit (Qiagen) and oligo-dT primers (Promega) according to the manufacturer’s protocol. Gene expression was analyzed by quantitative real-time PCR using TaqMan technology on a 7500/7500 FAST Fast Real-Time PCR instrument (Applied Biosystems). The following reagents were used: 5x QPCR Mix (Rox) from Bio&SELL, TaqMan Gene Expression Assays mouse IL-2 (Mm00434256_m1), mouse PKCθ (Mm01340226_m1) and mouse GAPDH Endogenous Control (4352339E) (both Applied Biosystems). All amplifications were conducted in duplicates. The housekeeping gene GAPDH was used for normalization.

### SDS Page and immunoblotting

Cell lysates from Jurkat T cells, HEK-293T cells or sorted murine CD4^+^ T cells were prepared and subjected together with precision plus protein standard (Bio-Rad) to SDS gel electrophoresis and immunoblotting as described previously [17]. Unspecific binding was blocked with 5% non-fat milk powder. Antibodies detecting PKCθ (27/PKCθ), actin (sc-1615) or Fyn (sc-16) were purchased from BD Biosciences and Santa Cruz Biotechnology, respectively. HRP-conjugated secondary antibodies against mouse, goat and rabbit IgG were purchased from Thermo Scientific. For detection, SuperSignal West Pico Chemiluminescent Substrate (Thermo Scientific) was used and the bands were visualized using hyperfilm (Amersham Biosciences) and Superfix Enviro Safe 25 together with Roentoroll Enviro Safe AC (Tetenal). Quantification of the Western blot bands was performed using Image J software.

### Protein stability assay

CD4^+^ T cells of wild type and PKCθ-E2^mut^ mice were treated with 50 μg/ml cycloheximide (dissolved in DMSO, both Sigma) for 6, 12, 16, 20 and 24 h and protein lysates were subjected for SDS Page and immunoblotting for actin and PKCθ. Quantification of the Western blot bands was performed using Image J software and a ratio of PKCθ to actin was calculated. Data are then depicted as percent of PKCθ/actin at t = 0 of each experimental group.

### Statistical analysis

The number of mice used per experiment and the number of experiments performed are listed in each figure legend. The data were analyzed for statistical significance by One sample t-test when comparing data presented as relative values to the control group or by one-way ANOVA with the Bonferroni post hoc test when comparing multiple samples within one graph. These statistical analyses were performed with GraphPad Prism software (GraphPad Software Inc.). A *p* value < 0.05 was considered statistically significant. Symbols used in the figures are: * *p* ≤ 0.05, ** *p* ≤ 0.01 and *** *p* ≤ 0.001.

## Results and discussion

### N-terminal V1 domain of PKCθ is essential for IL-2 transactivation in Jurkat T cells

While the N-terminus of conventional PKC isoenzymes contains the pseudosubstrate region, important for auto-inhibition, this region is rather variable in the subfamily of novel PKCs and has been implicated in isoenzyme-specific functions [10]. We addressed its relevance for PKCθ function by exchanging the sequence of exon 2, which encodes for the corresponding variable region (named V1). The sequence of the exon 2 replacing amino acids was chosen based on the PaptorX structure prediction program (http://raptorx.uchicago.edu). This E2^mut^ extension, albeit being a longer amino acid sequence than the wild type exon 2 encoded sequence, revealed to be the best fit of a highly unstructured domain not affecting the overall structure of the adjacent C2-like domain encoded in exon 3 of PKCθ. Of note, this E2-replacement mutation does not target the conserved C2-like domain and the tyrosine (Y) 90 residue within (Fig. 1a), whose phosphorylation by Lck regulates membrane translocation of PKCθ and downstream transcription factor activation events [7, 18]. Thus, the Y90F mutation impairs the ability of the catalytically active PKCθ-A148E mutant to induce NFAT as well as NF-κB activation. Additionally, the other five phosphorylation sites (T219, T538, S676, S68, and S695) that have been reported to play critical roles in the regulation of PKCθ function and downstream signaling (reviewed in 9) are still present in their wild type form in the PKCθ-E2^mut^ version (Fig. 1a). We tested the effect of the E2-replacement mutation in context of the constitutively active PKCθ mutant generated by the established Ala to Glu (A148E) exchange within the pseudosubstrate sequence to introduce a negative charge that mimics the presence of a phosphate at this location. This A/E background was used, since due to the high levels of endogenous PKC family member expression in Jurkat T cells, phorbol ester and ionomycin treatment to mimic TCR stimulation is not suitable to address the function of ectopically expressed PKCθ versions. Our experiments showed that, the E2-mutated PKCθ-A148E failed to induced IL-2 transactivation in transfected ionomycin-stimulated Jurkat T cells (Fig. 1b). The E2-mutation abolished also the capacity of the constitutively active PKCθ-A148E mutant to transactivate NFAT and AP-1 when assessed with a NFAT-AP1-dependent promoter luciferase reporter assay (Fig. 1c). However, due to endogenous PKCθ in Jurkat T cells, we could not convincingly show equal expression level of the ectopically PKCθ-A148E/E2^wt^ and PKCθ-A148E/E2^mut^ protein by immunoblot in transfected Jurkat T cells. Therefore, we decided to express a range of levels of PKCθ-A148E/E2^wt^ and PKCθ-A148E/E2^mut^ in Jurkat T cells and analyze the transfected cells in the NFAT-AP1-dependent promoter luciferase reporter assay. While we observed a dose-dependent increase of the luciferase signal when Jurkat T cells were transfected with PKCθ-A148E/E2^wt^, we did not detect signals above control transfected cells at any of the tested PKCθ-A148E/E2^mut^ plasmid concentrations (Fig. 1c), suggesting that the “normal” N-terminus is essential for PKCθ function in T cells.

In addition, we analyzed the activity of PKCθ-E2^wt^ and PKCθ-E2^mut^ not harboring the A148E mutation in cells that lack endogenous PKCθ expression. Therefore, HEK-293T cells were co-transfected with the NFAT-AP1-dependent promoter luciferase reporter construct and either the E2^wt^ or the E2^mut^ version of PKCθ. Expression of PKCθ-E2^mut^ impaired NFAT-AP1 transactivation in this cellular system, even though PKCθ-E2^wt^ and PKCθ-E2^mut^ protein expression was comparable when determined by immunoblot (Fig. 1d). Of note, however ionomycin/phorbol ester treatment seemed to activate endogenous kinases other than PKCθ since a strong activation of the NFAT-AP1-dependent promoter luciferase reporter construct was observed already in HEK-293T cells that were co-transfected with the empty control plasmid. Thus, ectopic expression of PKCθ-E2^wt^ only marginally increased the transactivation. Nevertheless, ectopic expression of PKCθ-E2^mut^ suppressed NFAT-AP1 transactivation, suggesting a dominant negative effect of the mutant on other PKCs. Altogether, in ectopic expression analysis in HEK-293T or Jurkat T cell lines, respectively, PKCθ-E2^mut^, when expressed at similar levels to the wt enzyme, shows a loss-of-function phenotype.

### Mice bearing the PKCθ-E2 mutation show reduced PKCθ expression on protein level

**Fig. 2:**
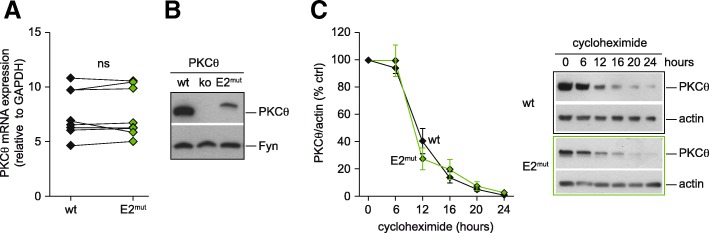
PKCθ-E2 mutation impairs PKCθ expression at the protein but not at the mRNA level. **a** PKCθ mRNA expression of MACS-sorted CD4^+^ T cells isolated from wt or E2^mut^ mice was analyzed by qRT-PCR. The house keeping gene gapdh was used for normalization. Each symbol represents values obtained of an individual mouse (*n* = 9), the connecting lines show indicate which samples were analyzed together in one experiment. Statistical significance was determined with paired t-test. **b** PKCθ protein expression of MACS-sorted CD4^+^ T cells from wild type (wt), PKCθ-deficient (ko) and mice expressing a PKCθ version with mutated exon 2 (E2^mut^) was analyzed by SDS-PAGE and immunoblot. Fyn was used as loading control. Representative results of one experiment (of total *n* = 8) are shown. **c** Representative western blots of PKCθ and actin expression levels of CD4^+^ T cells isolated from PKCθ-E2^wt^ and PKCθ-E2^mut^ mice untreated and after cycloheximide treatment for the indicated time points. The graph summarizes the results of two independent experiments with total of four experimental groups per genotype (mean ± SEM)

After having observed the importance of PKCθ’s N-terminus for T cell activation events in cell lines using overexpression of a mutant version of PKCθ, we wanted to confirm this finding under more physiological conditions in vivo. Therefore, a mouse line carrying the mutated version of exon 2 (PKCθ-E2^mut^) was generated by targeted mutation in embryonic stem cells. PKCθ-E2^mut^ mice were born following the expected Mendelian frequency with no obvious difference in viability and fertility, normal growth and weight development (unpublished observations). First, we analyzed the expression of the mutated version of PKCθ in T cells of the newly generated mouse line. At the mRNA level, quantitative real-time PCR did not reveal significant differences between the expression of wild-type (wt) and E2^mut^ PKCθ in isolated CD4^+^ T cells, (Fig. 2a). However, at the protein level, there was reduced expression of the PKCθ-E2^mut^ protein, which is 43 amino acids larger than its wild type counterpart (707aa; Fig. 2b). To address whether the E2 mutation impairs PKCθ protein stability, CD4^+^ T cells of wild type and PKCθ-E2^mut^ mice were treated for different times with cycloheximide, which inhibits protein synthesis. PKCθ and as internal control actin protein levels were examined by western blot analysis. Protein levels of both PKCθ versions strongly decreased between 6 and 12 h of cycloheximide treatment (in relation to untreated cells). However, no significant differences in protein turnover rate of PKCθ-E2^mut^ protein could be observed in these experiments (Fig. 2c).

### PKCθ-E2 mutation impairs T cell development in vivo

**Fig. 3:**
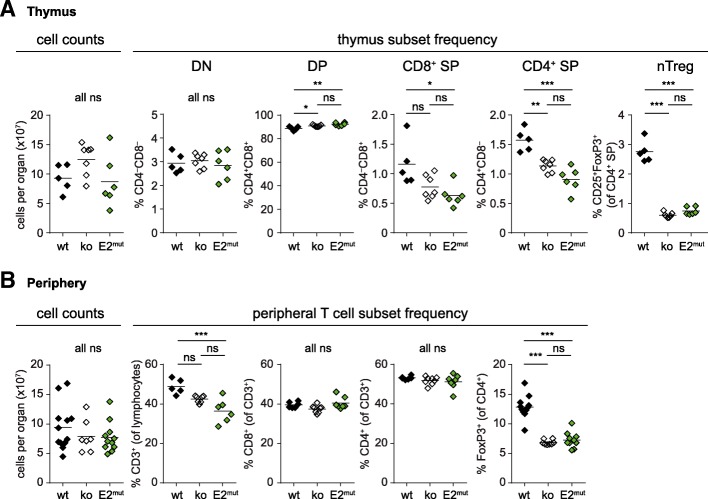
Similar changes in the T cell compartment of PKCθ-deficient and PKCθ-E2^mut^ mice. **a** Total thymocyte counts were determined ex vivo using a Luna cell counter. The frequency of thymocyte subsets (DN, DP, CD4^+^ and CD8^+^ SP as well as nTregs) was analyzed by flow cytometry. Graphs summarizing all experiments are shown (the gating strategy and representative FACS dot blots are shown in Additional file 1: Figure S1). Each symbol represents values obtained of an individual mouse (*n* ≥ 5), the solid line shows the mean value. DN = Double negative; DP = double positive; SP = single positive. **b** Cell counts of combined spleen/lymph nodes was determined by Luna cell counter and the frequency of T cell subsets was analyzed by flow cytometry (the gating strategy and representative FACS dot blots are shown in Additional file 2: Figure S2). In (**a**) and (**b**), results of at least 4 independent experiments with a total of 5 or more mice per genotype are shown. Statistical analyses were performed using one-way ANOVA and Bonferroni’s multiple comparison test

Next, we addressed whether mice expressing PKCθ-E2^mut^ instead of wt PKCθ show any abnormalities in their immune status, especially within the T cell compartment since it has been reported that positive selection during thymocyte development is affected in PKCθ-deficient mice [19, 20]. In line with these previous studies, we observed reduced frequencies of CD4 and CD8 single positive thymocytes in mice lacking PKCθ (Fig. 3a and Additional file 1: Figure S1), which was reflected by a decreased percentage of peripheral mature T cells (Fig. 3b). Even though CD3^+^ frequency among lymphocytes was lower in both genotypes it only reached statistical significance in case of PKCθ-E2^mut^ mice. Of note, PKCθ-E2^mut^ mice showed similar impairment in thymic development as PKCθ-deficient mice (Fig. 3a). Flow cytometric analyses of thymocytes showed that there was a strong and comparable reduction in Foxp3^+^CD25^+^CD4^+^ natural regulatory T cells (nTreg) in PKC-deficient and PKCθ-E2^mut^ mice as compared to wt mice. Correspondingly, a reduction in Treg frequency was also observed in secondary lymphoid organs of both the analyzed PKCθ genotypes (Fig. 3b and Additional file 2: Figure S2), which has been already published for PKCθ-deficient mice [21, 22]. Nevertheless, currently it is not clear whether Treg-intrinsic PKCθ expression or its expression by conventional T cells is critical for thymic Treg development. Both sound reasonable, since induction of Foxp3 expression and Treg differentiation require increased signal strength [23], which might be reduced when PKCθ is absent. However, Treg development also depends on IL-2 provided by T cells in the thymus [24], whose expression is reduced when CD4^+^ T cells lack PKCθ. In contrast to the altered Treg compartment, however, naïve/memory distribution among CD4^+^ as well as CD8^+^ T cells in the periphery of PKCθ-deficient and PKCθ-E2^mut^ mice was comparable to wt mice (Additional file 3: Figure S3). Furthermore, no gross changes in other immune cell compartments such as B cells, neutrophils and macrophages were detected (data not shown) as also no significant differences in overall spleen/lymph node cell counts (Fig. 3b).

### Reduced activation of CD4^+^ T cells expressing PKCθ-E2^mut^

**Fig. 4:**
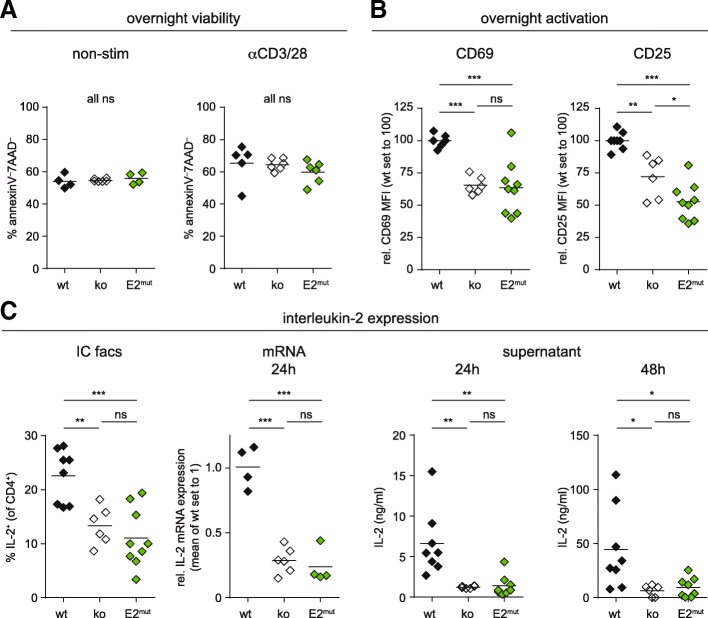
Early activation but not survival of PKCθ-E2^mut^ CD4^+^ T cells is impaired. **a** Viability of CD4^+^ T cells after overnight culture (total spleen/LN cells), either non-stimulated or stimulated with anti-CD3 and anti-CD28 antibodies, was determined by Annexin V/7AAD staining and flow cytometry. **b** CD25 and CD69 expression by CD4^+^ T cells of the three different genotypes was analyzed by flow cytometry upon overnight stimulation with anti-CD3 and anti-CD28 antibodies (total spleen/LN cells, gated on CD4^+^). The results are depicted as mean fluorescence intensity, MFI, relative to wt. **c** Interleukin-2 expression of CD4^+^ T cells of wt, PKCθ-deficient (ko) and PKCθ-E2^mut^ mice was analyzed by intracellular staining and flow cytometry (IC FACS, see Additional file 4: Figure S4 whole LN/spleen stimulated with anti-CD3/CD28 for 7 h, in the presence of GolgiPlug). IL-2 expression of MACS-sorted CD4^+^ T cells stimulated with anti-CD3 and anti-CD28 antibodies for the indicated time was analyzed by RT-PCR on mRNA level (normalized with gapdh and depicted relative to wt) and on protein level by luminex analysis of the supernatant. Each symbol represents values obtained on an individual mouse (*n* ≥ 6), the solid line shows the mean value. Statistical significance was determined by one-way Anova and Bonferroni’s multiple comparison test

Mice deficient in PKCθ not only show a defect in thymic positive selection, as mentioned above, but also have a severe impairment in peripheral CD4^+^ T cell activation, especially regarding IL-2 expression [3, 4]. Moreover, PKCθ has been implicated in T cell survival, which was reduced when T cells lacked PKCθ expression [25, 26]. In contrast, we observed comparable survival of CD4^+^ T cells regardless of their genotype by Annexin V/7AAD staining and flow cytometry after overnight in culture - either non-stimulated or stimulated with anti-CD3 and anti-CD28 antibodies (Fig. 4a). But unlike in the published studies, we first cultured the cells only overnight and not for 36 or 72 h and second, used non-sorted splenocytes instead of isolated CD4^+^ T cells and identified CD4^+^ T cells thereafter by flow cytometry. Nevertheless, we observed reduced upregulation of the early activation markers CD25 and CD69 upon stimulation overnight when T cells expressed the mutant form of PKCθ or lacked PKCθ expression (Fig. 4b).

Moreover, IL-2 expression of PKCθ-deficient as well as PKCθ-E2^mut^ CD4^+^ T cells was impaired at both the mRNA and protein level (Fig. 4c and Additional file 4: Figure S4). This finding is in agreement with publications reporting that exogenous IL-2, which acts as an extrinsic survival factor, did overcome the survival defects of PKCθ-deficient CD4^+^ T cells. Hence, we assume reduced viability secondary to lower IL-2 expression at later time points. Of note, the phenotype of PKCθ-E2^mut^ CD4^+^ T cells did resemble the defects in early activation response and IL-2 expression observed for PKCθ-deficient CD4^+^ T cells (Fig. 4b, c), supporting the importance of the N-terminal V1 domain for PKCθ-mediated functions. However, we cannot rule out that this phenotype is caused by reduced PKCθ protein amount (as shown in Fig. 2b). Nevertheless, the results obtained with CD4^+^ cells of PKCθ-E2^mut^ mice are in line with our findings with HEK-293T cells described above (Fig. 1d), where comparable expression of wt and E2-mutant PKCθ protein was detected.

## Conclusion

PKCθ is well known as an important kinase downstream of the TCR positively regulating T cell activation [3–8]; for instances early IL-2 expression depends on its function. This PKC isoenzyme shares a general domain structure with other members of the subfamily of novel PKCs; comprising the conserved C1a, C1b, C2-like and kinase domains (Fig. 1a). However, variable regions, which are distinct between novel PKC isoenzymes, seem to contribute to isoenzyme-selective functions [10] and might be also of importance for cell type specific actions of PKCs. For example, the proline-rich motif within the V3 domain is involved in interaction between PKCθ and CD28 [11] and the V5 domain contributes to enzyme activation by stabilization of the catalytic core structure [12]. The functional importance of the very N-terminal variable ‘V1 region’, which we investigated in our current study, has to the best of our knowledge not been analyzed so far. To address its role for PKCθ functions in CD4^+^ T cells an exon 2/V1-domain exchange mutant of PKCθ was ectopically expressed in cell lines and additionally, the T cell compartment of a genetically modified mouse line (PKCθ-E2^mut^) was analyzed. The PKCθ mutation resulted in replacement of the first 39 amino acids, avoiding targeting the integrity of conserved C2-like domain. The chosen E2^mut^ extension serves as an unstructured domain not affecting overall structure of the C2-like domain encoded in exon 3.

We observed that this exon 2 mutation of PKCθ impairs activation of T cells, such as the expression of IL-2, in human cell lines as well as in the genetically modified mouse line. Moreover, the importance of PKCθ for thymic development, especially the nTreg compartment, which has been described before using conventional PKCθ-deficient mice by us and others [21, 22], could be confirmed using our PKCθ-E2^mut^ mouse line. In the murine system, we observed reduced protein levels of the mutated version of PKCθ, resulting in a hypomorph mutant of PKCθ. As a caveat, however, we cannot exclude the possibility of a missfold contributing to the loss-of-function of PKCθ-E2^mut^ protein, even though the protein half-life of both PKCθ protein versions seemed to be comparable. We can only speculate, why so much less PKCθ-E2^mut^ protein is expressed in comparison to wild type PKCθ, since the differences in protein expression are not due to faster degradation. Possible explanations include impaired mRNA export from the nucleus as well as compromised translation initiation or elongation.

In summary, our results strongly indicate that mutation of exon 2 provides an alternative PKCθ loss-of-function mouse line that phenocopies the already published PKCθ-deficient mouse lines [3, 4]. Hence, this novel PKCθ-E2-mutant mouse line independently highlights the critical role of PKCθ downstream of the antigen-receptor complex for T cell development as well as T cell activation responses.

## Additional files

Additional file 1: **Figure S1.** (**a**) FACS dot plots depicting the gating strategy used for analyzing the thymic subsets are shown in figure 3A. (**b**) Representative FACS dot blots showing thymocyte subsets of all three genotypes (wt, PKCq-deficient and PKCq-E2mut mice). Corresponding quantification is presented in figure 3A. (EPS 1820 kb)

Additional file 2: **Figure S2**. (**a**) FACS dot plots depicting the gating strategy used for analyzing peripheral T cell subsets shown in figure 3B. (**b**) Representative FACS dot blots showing Treg staining of all three genotypes. Corresponding quantification is presented in figure 3B. (EPS 1560 kb)

Additional file 3: **Figure S3.** Normal peripheral T cell subsets in PKCq-deficient and PKCq-E2mut mice. (**a**) Frequencies of naïve and memory CD4+ T cells and (**b**) naïve, effector and central memory CD8+ T cells in the secondary lymphoid organs (spleen/LN) of the three genotypes (wt, PKCq-deficient and PKCq-E2mut mice) were analyzed by flow cytometry (using CD62L and CD44 as markers). Results of 4 independent experiments with a total of at least 5 mice per genotype are shown. Statistical analyses were performed using one-way ANOVA and Bonferroni’s multiple comparison test. (EPS 6680 kb)

Additional file 4: **Figure S4.** Representative FACS dot blots showing intracellular IL-2 staining of CD4+ T cells from all three genotypes along with one unstimulated wt sample. Corresponding quantification is presented in figure 4C. (EPS 1140 kb)

## Abbreviations

AP-1: Activating protein 1
CA: Constitutively active
DAG: Diacylglycerol
E2^mut^: Exon 2-mutation
IL-2: Interleukin-2
IS: Immunological synapse
NFAT: Nuclear factor of activation in T cells
NF-κB: Nuclear factor κ B
PDBu: Phorbol 12,13-dibutyrate
PKC: Protein kinase C
TCR: T cell receptor
